# Physiological and pathophysiological functions of NLRP6: pro- and anti-inflammatory roles

**DOI:** 10.1038/s42003-022-03491-w

**Published:** 2022-06-01

**Authors:** Diego Angosto-Bazarra, Cristina Molina-López, Pablo Pelegrín

**Affiliations:** 1 Línea de Inflamación Molecular, Instituto Murciano de Investigación Biosanitaria IMIB-Arrixaca, Hospital Clínico Universitario Virgen de la Arrixaca, 30120 Murcia, Spain; 2 Department of Biochemistry and Molecular Biology B and Immunology, Faculty of Medicine, University of Murcia, 30120 Murcia, Spain

**Keywords:** Immunology, Cell biology

## Abstract

The nucleotide-binding oligomerization and leucine-rich repeat receptor (NLR) protein family consists of important immune sensors that form inflammasomes, a cytosolic multi-protein platform that induces caspase-1 activation and is involved in different inflammatory pathologies. The NLR family pyrin domain containing 6 (NLRP6) is a receptor that can signal by forming inflammasomes, but which can also play an important role without forming inflammasomes. NLRP6 regulates intestinal homeostasis and inflammation, but also is involved in cancer, the nervous system or liver diseases, with both protective and deleterious consequences. In the present article, we review the different roles of NLRP6 in these processes and offer new insights into NLRP6 activation.

## Abstract (web summary)

This review discusses emerging roles for the NLR family pyrin domain containing 6 receptor (NLRP6) in intestinal homeostasis, inflammation, cancer, the nervous system and liver disease.

## Introduction

Since the first inflammasome was described as a multiprotein cytosolic oligomer for caspase-1 activation and interleukin (IL)-1β processing^1^, these oligomers have played an increasingly prominent role in human pathology. Inflammasomes are important in autoimmune syndromes, chronic inflammatory diseases, and metabolic and degenerative pathologies^2–6^. The activation of inflammasomes is related to the cellular sensing of different molecular patterns associated with pathogens (PAMPs), cellular damage or death (DAMPs), microbes (MAMPs) or homeostasis-altering processes (HAMPs)^7–12^ (Table 1). These signals are sensed by intracellular nucleotide-binding oligomerization and leucine-rich repeat receptors (NLRs) that form oligomers through homotypic interactions with the apoptosis-associated speck-like protein containing a caspase activation and recruitment domain (ASC). ASC forms oligomeric filaments and functions as the adapter molecule between the oligomer of NLR and the pro-inflammatory effector protease caspase-1. Caspase-1 is auto-proteolytically activated after recruitment and cleaves to pro-inflammatory cytokines of the IL-1 family, such as IL-1β and IL-18. Caspase-1 also releases the N-terminal domain of gasdermin D (GSDMD), which binds to the inner plasma membrane to form oligomeric pores with size and charge that facilitate the release of the processed IL-1β and IL-18. If GSDMD pores in the plasma membrane are not repaired, the plasma membrane ninjurin-1 protein induces pyroptotic cell death and massive intracellular content release^13,14^.

**Table 1:** Modulators of NLRP6 inflammasome.

Type signal	Signal	Reference
PAMPs	Lipopolysaccharide (LPS)*	^31^
	Lipoteichoic acid (LTA)*	^7,43^
	viral dsRNA*	^28,43,57^
MAMPs	Commensal microbiota*	^20,45^
	Parasite commensal*	^49,50^
	Periodontal pathogens*	^75^
Other modulators of NLRP6	Taurine*	^46^
	Spermine**	^46^
	Histamine**	^46^
	Nicotine*	^93^

(*) Activator

(**) Inhibitor

The NLR proteins contain an N-terminal domain that could be either a pyrin domain (PYD) or a caspase activation and recruitment domain (CARD) leading to the NLRP and NLRC sub-families respectively. NLR members also include a central neuronal apoptosis inhibitory protein NAIP, a major histocompatibility class II transcription activator CIITA, an incompatibility locus protein from *Podospora anserina* HET-E, a telomerase-associated protein TP1 (NACHT) domain, and a C-terminal leucine-rich repeat (LRR) domain. The NLR family includes several members that form inflammasomes and because of the large number of members discovered (23 in humans and more than 30 in mice^15^) they have been associated with multiple functions of the innate immune system.

NLRP6, as an NLR family member^16^, covers both inflammasome-dependent and independent functions by activating caspase-1 or caspase-11 or regulating the key transcription factor NF-κB^17,18^. NLRP6 has an important role in maintaining intestine homeostasis, and more specifically in regulating the interaction between the host mucosa and the microbiota^19,20^. Other physiological roles played by NLRP6 are emerging, such as its role in helping the host to defend itself against pathogens, in tumorigenesis, and in neuroinflammation^21–23^, where NLRP6 presents pro- and anti-inflammatory roles. The fact that NLRP6 has differential roles depending on the tissue where it is expressed and activated makes this receptor an exciting and complex new therapeutical target. This review aims to summarize the inflammasome and non-inflammasome-mediated roles of NLRP6 in health and disease.

## New insights into NLRP6 transcription

NLRP6 is highly expressed in the digestive system^20,24^ and most of the functional studies of NLRP6 focus on this system and its related diseases. However, NLRP6 is also expressed in other tissues such as the kidney, liver and lung and in cells such as neurons, lymphocytes, or bone marrow-derived cells^7,20,25^. *NLRP6* is a gene with multiple alternative transcriptional promoters that determine its distribution and expression across selective tissue and cell types^24^. In humans, *NLRP6* has three alternative transcription starting sites (TSS), the first is located exon 1 (in the 5′ untranslated region), which is selective for intestine expression, the second is located in exon 2 (in the middle of the PYD domain), which is selective for expression in kidney, lung, liver, spleen, and brain, and the third is located in exon 3 (in the region between PYD and NACHT domains)^24^. All three TSS are present in the kidney, although its predominant transcription starting site is the second (exon 2), meaning that the *NLRP6* detected there is truncated. In contrast, in the small intestine, the predominant transcription starting site is the first (exon 1), meaning that in this case, the NLRP6 protein is endogenous, full-length, and highly detectable. Therefore, the translational repression of human NLRP6 occurs in a tissue-specific context (outside the intestinal epithelium) and is the result of alternative promoters^24^. In mouse, the *Nlrp6* gene is expressed in kidney, liver, and intestinal tissue; however, only endogenous NLRP6 protein was detected in the intestine^24^. This is due to two distinct alternative promoter isoforms of *Nlrp6*, one expressed in the intestine (in the exon 1 containing the canonical 5′ untranslated region of 185 bp) and the other expressed in the kidney and liver with an expanded 5′ untranslated region sequence of 1749 bp that results from complex alternative splicing comprising the exon 1 of *Nlrp6*, two upstream intergenic exons and the exon 1 of the adjacent upstream gene *BC024386* (expressed in kidney and liver but not in the colon), thus generating an alternative promoter^24^. This suggests that mouse *Nlrp6* can be regulated by tissue-selective alternate promoters that result in two isoforms, one for the expression in the intestine and the other for the expression in the kidney and liver^24^. In this regard, it has been shown in a mouse model of nephrotoxic acute kidney injury (AKI) induced by a folic acid overdose that NLRP6 is downregulated in the kidney^26^.

Mapping of transcription factor binding sites using JASPAR and TRANSFAC databases revealed that the *Nlrp6* gene in mouse presents overrepresented peroxisome proliferator-activated receptor γ-retinoid X receptor-α (PPARG-RXRA) binding sites in its promoter region, which are also conserved in human and rat^27^. In this regard, human epithelial cells from colon tissue treated with rosiglitazone, a PPAR-γ agonist, showed an increase in NLRP6 gene expression, which suggests that modulators of PPAR-γ could be a target to control *Nlrp6* transcription^27^.

Viral infections and the production of interferon type I/III upregulate *Nlrp6* gene expression in mouse intestine and in fibroblast^28^, which suggests that NLRP6 plays a role in the host response to viral infection. This role is specifically important in the intestinal tract where Nlrp6 limits the ssRNA virus replication by interacting with the Asp-Glu-Ala-His (DEAH) box helicase 15 (Dhx15)^28^. The transcriptional expression of mouse *Nlrp6* has been found to be negatively regulated by the miR-33-3p in microglial cells treated with hemin in a model of intracerebral hemorrhage^29^. Another microRNA involved in the downregulation of *Nlrp6* is miR-650, which targets the 3′-untranslated regions of *Nlrp6* and thus inhibits its expression in ulcerative colitis^30^. Therefore, the complexity that NLRP6 shows in its transcriptional regulation could reveal different roles for NLRP6 that need to be clarified in other tissues besides the intestine, liver, lungs, or kidney.

## Structural assembly of the NLRP6 inflammasome

**Fig. 1:**
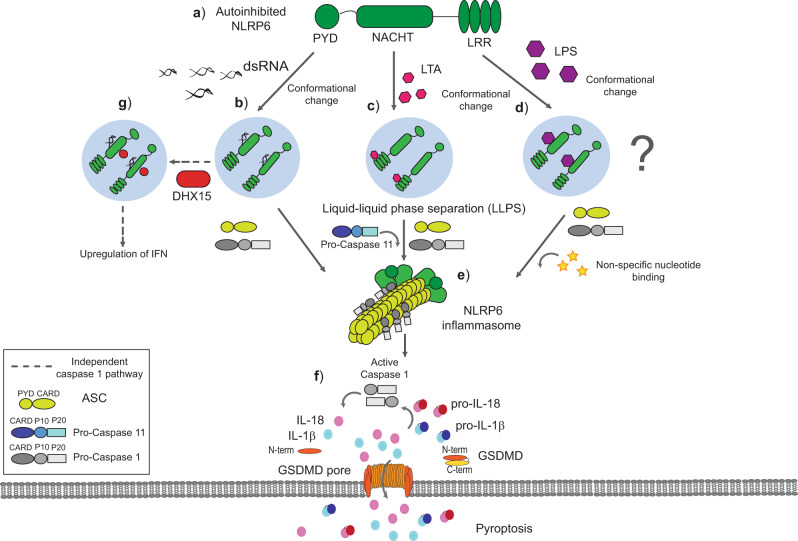
New insights into NLRP6 inflammasome formation. **a** The NLRP6 protein is autoinhibited in basal conditions. **b**, **c** dsRNA, LTA can bind directly to NLRP6, thus causing a possible conformational change to help LLPS as an early step necessary for inflammasome formation. **d** LPS can bind directly to NLRP6, which probably leads to the formation of LLPS, although this is not known; in vitro recombinant NLRP6 protein requires ATP after LPS binding to form an oligomer. **e** If ASC binds to NLRP6 oligomer, it induces a solid particle of inflammasome **f** that activates caspase-1 and/or caspase-11, which in turn processes GSDMD and results in plasma membrane pore formation and the release of pro-inflammatory cytokines and intracellular content. **g** If the NLRP6 inflammasome is not formed by interaction with ASC, NLRP6 in LLPS induces an alternative inflammasome-independent pathway by inducing interferon and interferon-stimulated genes^43^.

Once NLRP6 has been expressed, inflammasome formation and activation is mediated by the interaction between an inflammasome sensor protein and the adapter protein ASC; however, this binding requires the sensor protein to form an oligomer with the PYD domains in order to seed ASC. By studying cryo-EM and crystallography structures Shen et al.^31^ demonstrated that the PYD domain of NLRP6 alone can self-assemble into a filamentous structure that can bind to ASC through homotypic PYD-PYD domain interactions. The interaction between ASC and the PYD of NLRP6 is enhanced when the central domain NACHT of NLRP6 is fused to its PYD domain. Their study suggests that the PYD domain of NLRP6 could seed the inflammasome assembly with the PYD domain in the center and surrounded by the NACHT and LRR domains^31^, similarly to other described inflammasomes such as NLRP3^32^. These studies imply that NLRP6 undergoes a conformational change to allow the interaction of ASC and therefore suggest that inactive NLRP6 should adopt an auto-inhibited state (Fig. 1a). The active NLRP6 conformation could be triggered by different stimuli, including direct binding of ligands to NLRP6^33,34^ (Table 1). In this regard, lipopolysaccharide (LPS), the major component of the outer membrane of gram-negative bacteria, can directly bind to recombinant purified NLRP6 in vitro, thus producing the conformational change and the subsequent homodimerization of NLRP6^35^. In cellular assays, intracellular delivery of LPS also induced colocalized foci between NLRP6 and ASC when these proteins were ectopically expressed. In vitro, the recombinant purified NLRP6 requires ATP to interact with ASC after LPS binding and form an oligomer (Fig. 1e). Similar to LPS, lipoteichoic acid (LTA), a component of gram-positive bacteria surface, can also bind to NLRP6 through the LRR domain and promote ASC oligomerization (Fig. 1d). Functionally, NLRP6 activation by LTA activates caspase-11 rather than caspase-1^7^. Caspase-11 is then able to activate the non-canonical pathway of the NLRP3 inflammasome by inducing cellular K^+^ efflux via GSDMD pores. The subsequent activation of the NLRP3 inflammasome activates caspase-1 and IL-1β maturation^7,36,37^.

Recently, the action of liquid-liquid phase separation (LLPS) has been found to be associated with NLRP6 activation, and this phenomenon is gaining interest in the study of physiology and disease. Macromolecules such as proteins undergo LLPS when they condense into a dense phase that coexists with a diluted phase^38^. This LLPS has been related to neurodegenerative diseases, cancer, viral infections, and the immune response^39–42^. In a recent study, LTA and viral dsRNA have been described as promoting NLRP6 to form LLPS (Fig. 1b–d), which questions the paradigm that NLRP6 active inflammasomes form structured oligomers that promote activation^43^. The polybasic region of NLRP6 located in the NACHT domain (350-354) is required for LLPS^43^. After phase separation, the presence of ASC will solidify NLRP6 condensates and will lead to the activation of the inflammasome^43^ (Fig. 1f). This model proposes a novel mechanism of NLRP6 inflammasome activation in line with the single cellular big speck formed by NLRP6 harboring the PYD and interdomain of NLRP3^44^. These studies shed light on how NLRP6 inflammasome could be assembled and highlight the pleiotropic nature of NLRP6 singling.

## Function of NLRP6 in the digestive system

### NLRP6 in microbiota homeostasis

**Fig. 2:**
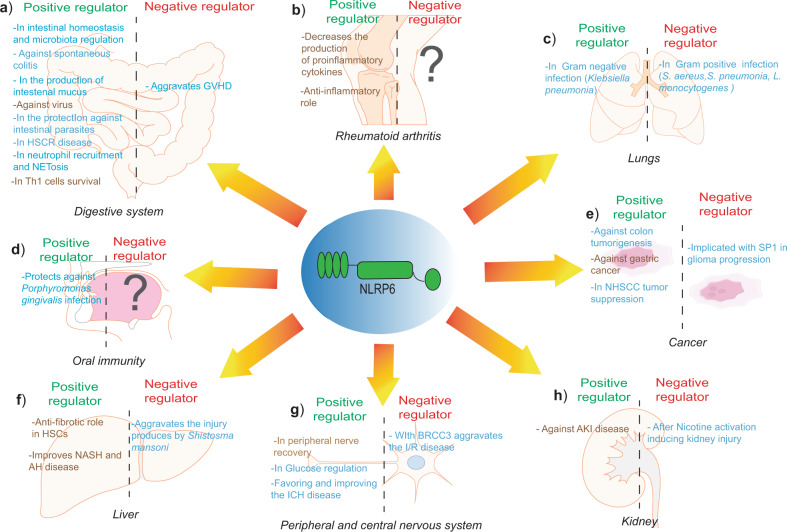
Roles of NLRP6 in different organs and tissues. Schematic representation of the different functions of NLPR6 in **a** the digestive system, **b** joints, **c** lungs, **d** oral cavity, **e** cancer, **f** liver, **g** peripheral and central nervous system, and **h** kidney. Functions shown in blue represent the NLRP6 inflammasome-dependent response and functions shown in brown represent the NLRP6 inflammasome-independent response. Illustrations were partially created using templates from www.motifolio.com (Motifolio Inc, Ellicott City, Md).

NLRP6 is highly expressed in the intestine, where it plays an important role in controlling intestinal homeostasis. It has been shown that a deficiency of NLRP6 affects the microbiota composition and that *Nlrp6*-deficient mice are more susceptible to chemically induced or spontaneous colitis associated with an increase in *Akkermansia muciniphila* in the gut, a specific pathobiont sufficient for promoting intestinal inflammation^20,45,46^ (Fig. 2a). The altered microbiome obtained by these *Nlrp6*-deficient mice after DSS-induced colitis was transferable to wild-type mice and made them more susceptible to DSS colitis, which in turn was followed by an increase in IL-6 signaling in intestinal epithelial cells, thus enhancing colorectal cancer formation^20,23,47^. In this regard, the dietary flavone apigenin improved chemically induced colitis in mice through the stress-induced regenerating islet-derived protein 3 (Reg3b), an immunomodulatory c-type lectin whose expression is regulated by NLRP6^48^.

Recent studies showed that the lack of NLRP6 in mice infected with the intestinal parasite *Cryptosporium* or with *Candida albicans* meant that these mice exhibited a higher parasite burden compared with wild-type mice due to a deficiency in the release of pro-inflammatory IL-18 and IL-1β, which are important cytokines that control the expansion of these parasites^49,50^ (Fig. 2a). NLRP6-dependent IL-18 release after *Cryptosporium* or *C. albicans* infection occurs via the canonical inflammasome pathway because it requires ASC, caspase-1, and GSDMD^49,50^. Different metabolites such as taurine, carbohydrates, and long-chain fatty acids are known as activators of NLRP6, which in turn reduces DSS colitis, whereas histamine and spermine are reported to be the suppressor of the NLRP6 inflammasome, which in turn exacerbates the DSS-induced colitis^46^. This suggests that the colitogenic microbiota are able to regulate the production of antimicrobial peptides (AMP) by shaping the host-microbiome interface and the susceptibility to DDS-induced colitis^46^. This suggests that intervening in the metabolite-inflammasome-antimicrobial peptide axis in order to treat intestinal diseases could be an exciting area of future search. In this regard, the upregulation of *Nlrp6* in infiltrating monocytes after DSS-induced colitis reduces the susceptibility to chemically induced intestinal injury by restoring the intestinal barrier and limiting the induction of bacteria-driven inflammation^51^. These data suggest that the microbiota composition and function may be related to the therapeutic modulation of NLRP6 function.

The intestinal epithelium is separated from most of the microorganisms by an intestinal mucus layer that is the primary defense against infections^52–54^. NLRP6 in goblet cells has been associated with a crisis in mucus production after the bacterial invasion, where bacterial Toll-like receptor (TLR) ligands stimulate the secretion of Muc2 through NLRP6 activation^19,55^ (Fig. 2a). However, the role of NLRP6 in the baseline inner mucus layer formation is not clear since *Nlrp6*^−/−^ mice presented a functional inner mucus layer^56^.

NLRP6 was reported to limit the replication of RNA viruses in the intestine^28,57^ (Fig. 2a). NLRP6 can bind through its NACHT domain to the Asp-Glu-Ala-His (DEAH) box helicase 15 (DHX15), which increases antiviral gene expression and promotes the interaction of ASC and NLRP6, thus indicating that DHX15 promotes the assembly of NLRP6 inflammasome and induces inflammasome activation and IL-18 production (Fig. 1g)^28,43,57^. However, certain viruses, such as the hepatitis virus, may activate the NLRP6 inflammasome independently of DHX15^43^.

In addition to epithelial cells, NLRP6 is also expressed in different cells of the innate and adaptive immune system, including T cells, monocytes, and neutrophils^7,22,25,51^. In Th1 cells, NLRP6 expression is controlled by the transcription factor TBX21, where it facilitates T cell survival^25^ (Fig. 2a). In neutrophils, NLRP6 controls NETosis, a specific type of cell death that depends on GSDMD and that releases decondensed chromatin and granular content into the extracellular space, that forms neutrophil extracellular traps (NETs) in order to eliminate bacterial infections^58,59^ (Fig. 2a). NETs are also involved in the development of intestinal inflammatory disease^58^. NLRP6 expressed in Ly6C^hi^ monocytes and neutrophils in the intestinal lamina propria controls tumor growth in the inflamed intestine via the production of IL-18^51^.

### NLRP6 in inflammatory intestinal diseases and gastric cancers

The NLRP6 has been associated with different inflammatory intestinal diseases such as ulcerative colitis or Crohn’s disease^60,61^. The expression of NLRP6 has been shown to be important for protection against inflammation-related colon tumorigenesis (Fig. 2e) since NLRP6-deficient mice develop a severe inflammatory state induced by upregulation of the cytokine C-C motif chemokine ligand 5 (CCL5) that together with altered microbiota produces IL-6, thus resulting in enhanced epithelial cell proliferation and inflammation-induced colorectal cancer^20,23,47^.

The ubiquitin carboxyl-terminal hydrolase CYLD prevents excessive IL-18 production by deubiquitinating NLRP6 and impairing inflammasome formation, thus preventing excessive inflammation^62^. Furthermore, in patients with ulcerative colitis elevated levels of IL-18 inversely correlate with CYLD expression, suggesting that CYLD deubiquitination of NLRP6 could be a new therapeutic approach for treating intestinal inflammation^62–64^.

Less studied is the role of NLRP6 in graft-versus-host disease (GVHD), where host NLRP6 aggravates gastrointestinal symptoms of GVHD and the above-mentioned taurine and the inflammasome action contribute to GVHD mortality^65^(Fig. 2a).

In gastric cancer, NLRP6 can bind through its PYD domain to the substrate-binding domain (SBD) of the 78 kDa glucose-regulated protein (GRP78), which in turn promotes the ubiquitination and degradation of GRP78 and thus reduces its expression and inhibits gastric cancer growth^66^. In line with the importance of NLRP6 in gastric cancer, another recent study shows that a long non-coding RNA named OIP-AS1 interacts with the zeste homolog 2 (EZH2), which is part of an essential polycomb repressive complex 2 whose deregulation is associated with different diseases, especially cancer, and contributes to gastric cancer cell growth and migration due to epigenetic silencing of NLRP6 transcription^67,68^ (Fig. 2e).

The role of NLRP6 has been also studied in individuals with Hirschsprung’s associated enterocolitis (HAEC), which is the principal cause of death in patients with Hirschsprung’s disease (HSCR), a congenital disorder characterized by the absence of ganglion cells at the end of the bowel^69,70^(Fig. 2a). NLRP6 expression is reduced in the colon of patients with HSCR compared with healthy controls, suggesting that the decreased expression of NLRP6 contributes to the formation of an altered microbiome due to the ineffective clearance of bacterial pathogens, thus making these patients more susceptible to developing HAEC^70^.

## NLRP6 in rheumatoid arthritis

In autoimmune rheumatoid arthritis disease, there is decreased expression of NLRP6 in fibroblast-like synoviocytes, and in vitro NLRP6 silencing resulted in high expression of IL-6 and IL-1β^18^. NLPR6 could therefore ameliorate the exacerbated production of pro-inflammatory cytokines in rheumatoid arthritis patients, acting as a docking site to facilitate the interaction of pivotal proteins for NF-κB activation (such as TAB2/3 with tripartite motif 38) and induce their degradation^18^ (Fig. 2b). Therefore, in synoviocytes, NLRP6 acts with a non-inflammasome function and inhibits the NF-κB transcription factor, and thus plays an anti-inflammatory role.

## Function of NLRP6 in the lung

During lung infection, NLRP6 on the one hand exacerbates inflammation induced by the gram-positive *S. aureus*^71,72^, and on the other, it reduces bacterial load and increases survival after infection with the gram-negative *Klebsiella pneumonia* controlling NETosis through of C-X-C motif chemokine ligand 1 (CXCL1*)*^73^ (Fig. 2c).

Specifically, LTA from *S. aureus* induces NLRP6 inflammasome activation and loss of neutrophils by pyroptosis and NETosis, thus increasing host mortality^71,74^. NLRP6 also limited ROS production and INF-γ after infection^71,72^, which suggests that blocking NLRP6 could be a therapeutical approach for augmenting neutrophil-associated bacterial clearance and improving pneumonia.

## NLRP6 in oral immunity

NLRP6 has a role in oral immunity since it is highly expressed in the human gingival tissue and is specifically activated in infected gingival fibroblasts with *Porphyromonas gingivalis*, a pathogen associated with chronic periodontitis, thus inducing pyroptosis and IL-1β and IL-18 release through NLRP6 inflammasome activation^75^ (Fig. 2c). However, the exact mechanism by which *P. gingivalis* activates NLRP6 in gingival fibroblasts remains unknown.

## NLRP6 in cancer

In addition to its aforementioned role in gastrointestinal tumors, NLRP6 is also implicated in other types of cancers such as head and neck squamous cell carcinoma (NHSCC), a type of cancer that is developed from the mucosal epithelium in the oral cavity^76^. Patients with NHSCC present increased expression of *NLRP6*, *IL1B*, and *GSDME* compared with healthy donors^77^. This high expression of *NLRP6* has been associated with a higher survival rate, which suggests that *NLRP6* plays a beneficial role as a tumor-suppressing gene in NHSCC^77^ (Fig. 2e).

In glioma, the transcriptional upregulation of NLRP6 is promoted through its binding with the transcription factor specificity protein 1 (SP1)^78^. In cell lines derived from malignant glioma, the genetic downregulation of SP1 decreased the migratory and invasive potential of these cells. This SP1 downregulation also reduced the expression of inflammasome components such as ASC, caspase-1, and IL-1β, which were restored after NLRP6 overexpression. This result suggests that SP1 and NLRP6 positively regulate the malignant behaviors of glioma cells and the growth of tumors^78^ (Fig. 2e).

In other types of cancer, such as skin cutaneous melanoma, the low expression of NLRP6 is associated with poor prognosis^79^.

## Function of NLRP6 in the liver

The role of NLRP6 in the liver has been widely reported. Both the lack of NLRP6 and its downregulation are related to increased liver injury and the progression of different liver diseases such as liver fibrosis, non-alcoholic steatohepatitis (NASH), and alcoholic hepatitis (AH)^80–85^. NLRP6 has an antifibrotic role in the liver because it decreases the levels of the main enzymes responsible for the degradation of the liver matrix, such as MMP2 and MMP9^80,81^. Furthermore, NLRP6 is downregulated during NASH and alcoholic hepatitis, thus increasing the pro-inflammatory and profibrotic effects of NF-κB, promoting fibrosis, and increasing the severity of the diseases^82,83^. In particular, in NASH NLRP6 controls the expression of CD36, which interferes with the accumulation of triglycerides^82^. In alcoholic hepatitis, NLRP6 is associated with the inhibition of the chemokine C-C motif ligand 20 (CCL20), which is responsible for activating the hepatic stellate cells involved in fibrosis^83^ (Fig. 2f). Also, NLRP6 dampens leukocyte infiltration during acute liver injury and protects the liver^20,46,84^. During allogeneic hematopoietic stem cell transplantation, NLRP6 protects against liver injury by inhibiting NF-κB signaling, thus reducing inflammatory cell infiltration and liver fibrosis^85^.

The functions of NLRP6 have been also linked with liver infections, specifically with *Shistosoma mansoni*, which is a parasite that causes granulomatous inflammatory reactions^86,87^. In contrast to the protective role that NLRP6 plays in NASH, alcoholic hepatitis, and allogeneic transplantation, in this case, it contributes to the formation of hepatic granuloma and aggravates the injury produced by this specific parasite (Fig. 2f), a fact that has been confirmed by the enhanced production of the anti-inflammatory cytokine IL-10 in spleen cells from *Nlrp6*^−/−^ infected mice followed by reduced production of CCL2, CXCL1, and CCL in the liver^87,88^.

## NLRP6 in the nervous system

### Function of NLRP6 in the peripheral nervous system

The role of NLRP6 in the peripheral nervous system (PNS) is becoming more apparent in the context of sterile- and bacterial-induced inflammation, with NLRP6 being involved in inflammasome-independent neuronal recovery from peripheral nerve injury by limiting the activation of extracellular signal-regulated kinase (ERK)^21^ (Fig. 2g). On the other hand, NLRP6 inflammasome can activate caspase-11, thus inducing a loss of enteric-associated neurons^89,90^. During enteric infections, NLRP6 inflammasome formation in excitatory enteric neurons is involved in neuronal loss and a subsequent reduction of gut motility^90^. After microbiota depletion, there is an NLRP6-dependent loss of specific enteric neurons involved in glucose regulation, which results in an increase in blood glucose^89^ (Fig. 2g).

### NLRP6 in the central nervous system

In the brain, NLRP6 functions as an inflammasome during ischemia/reperfusion (I/R) injury and intracerebral hemorrhage (ICH)^91,92^, but with opposite effects. In ICH NLRP6 is upregulated in perihematomal brain tissue via TLR4 and ameliorates brain injury, thus reducing brain water content, pro-inflammatory cytokines, NF-κB activity, and neurological deficit scores^91^. This result is supported by the fact when the miR-331-3p is overexpressed, it negatively regulates the transcription of *Nlrp6*, which reduces recovery of the neurological functions^29^. However, in I/R injury, the NLRP6 inflammasome is formed by interaction with the deubiquitinase BRCA1-BRCA2-containing complex subunit 3 (BRCC3) and promotes increased infarct volume and brain water content, together with decreased neurological scores and increased IL-1β^92^ (Fig. 2g).

## Function of NLRP6 in the kidney

In vivo assays of the kidney after unilateral ureteric obstruction (UUO) and nephrotoxic serum (NTS) administration suggest that NLRP6 is dispensable after kidney injury, given that no phenotype is observed in *Nlrp6*^−/−^ mice and no Nlrp6 protein is detectable in the kidney^24^. In kidney injury produced by UUO or NTS administration, NLRP6 seems to play little role in recovery; however, after AKI, its reduced expression and protein levels actually seem to cause or increase AKI^24,26^. NLRP6 attenuated nephrotoxic AKI, renal inflammation and infiltration of inflammatory cells (macrophages and neutrophils), and decreased fibrosis, meant that NLRP6 plays a nephroprotective role in the kidney^26^ (Fig. 2h). However, in the nicotine-induced kidney damage model, where nicotine acted as an NLRP6 inflammasome activator, NLRP6 increased kidney injury by increasing release of ASC, active caspase-1, and IL-1β^93^ (Fig. 2h). Kidney disease leads to high blood pressure^94^. NLRP6/angiotensin-vasopressin receptor (AVR) and adrenomedullin loci have been related to hypertension^95^, with NLRP6 being a proposed receptor for AVR^96^.

## NLRP6 and platelet formation

In a high-powered genome-wide meta-analysis of more than 66,000 individuals, NLRP6 was associated with variations in mean platelet formation (MPV) and platelet count (PLT)^97^. NLRP6 was included in the 68 genomic loci reliably associated with variations in MPV and PLT. The *NLRP6* gene is placed beside the *PSMD13* gene in the human chromosome 11, so these two genes could form a segregation tandem for MPV and PLT, meaning that further experiments are required in order to determine if NLRP6 plays a functional role in platelet formation.

## Conclusion

Inflammasomes are highly important as immune sensors due to their role in different pathologies; however, the NLRP6 inflammasome possesses the particular feature of having inflammasome-independent roles and is thus detrimental or beneficial depending on the pathology. In particular, it functions as an inflammasome by exacerbating inflammation and thus contributing to the elimination of intestinal parasites, viruses, and gram-negative bacteria. Also, the activation of NLRP6 inflammasome controls the growth of different types of cancer such as NHSCC, it is implicated in glucose regulation and it promotes the healing of brain injuries after intracerebral hemorrhages. However, the NLRP6 inflammasome does not play a beneficial role in gram-positive infections, GVHD, ulcerative colitis, glioma, ischemia/reperfusion brain injury, or liver infections with parasites. For the inflammasome-independent function, NLRP6 has a protective role in the regulation of NETosis after pneumonia infection, in gastric cancers, in liver diseases, rheumatoid arthritis, certain acute kidney injuries, and intestinal colitis. The recent advances that have shown how NLRP6 is assembled by phase separation will help to tackle the design of drugs to control NLRP6 activation. Drugs affecting the assembly and activation of NLRP6 by directly binding to it and altering its conformation could alleviate or mitigate the different adverse effects when the uncontrolled activation of this inflammasome occurs after gram-positive bacterial infection, GVHD, parasite liver infections, ischemia/reperfusion injury, or glioma. The fact that different metabolites can affect NLRP6 activation opens up the exciting possibility of new methods of metabolite-mediated immune modulation that will improve the treatment of intestinal inflammatory diseases.
